# Microbiome Analysis and Pharmacovigilance After Inhaled Glucocorticoid: Oral Dysbiosis With the Isolation of Three *Rothia* Species and Subsequent Sjögren’s Syndrome

**DOI:** 10.3389/fphar.2022.636180

**Published:** 2022-04-01

**Authors:** Przemysław Zdziarski, Mariola Paściak, Andrzej Gamian

**Affiliations:** ^1^ Department of Clinical Immunology, Lower Silesian Oncology Center, Wroclaw, Poland; ^2^ Military Institute WITI, Wroclaw, Poland; ^3^ Department of Immunology of Infectious Diseases, Hirszfeld Institute of Immunology and Experimental Therapy, Polish Academy of Sciences, Wroclaw, Poland

**Keywords:** inhaled glucocorticoids, *Rothia*, adverse drug reaction, microbiome and dysbiosis, Sjögren’s syndrome, macroglossia, epithelitis, autoimmunity

## Abstract

**Background:** Treatment of respiratory tract diseases with inhaled glucocorticoids is a form of therapy that has been used for many years. It shows lower potency of side effects; nevertheless, microbiome change, sinopulmonary dysbiosis, secondary immunodeficiency, and immunomodulatory effects are underestimated. The latest guideline recommendations introduce the use of empirical antibiotic and/or multiplying inhaled glucocorticoids in therapeutic intervention of asthma and chronic pulmonary obstructive disease.

**Aims and objectives:** The aim of the study was to describe a simple, universal, and cost-effective method of microbiome analysis for clinical trials. Such a general method for monitoring pharmacovigilance should be widely available and reliable.

**Methods:** The study material included two kinds of swabs, taken from the same mouth ulcerations of patients with asthma treated with a temporary quadruple dose of fluticasone. The microbiological investigation was performed, and identification of the isolates was carried out using the matrix-assisted laser desorption ionization–time of flight mass spectrometry (MALDI-TOF-MS) Biotyper.

**Results:** The analysis of dry swab demonstrated the presence of typical oral bacteria (*Neisseria* spp. and *Streptococcus* spp.), alongside with the potentially pathogenic *Actinomyces* spp. and three different *Rothia* species, identified simultaneously: *R. aeria*, *R. dentocariosa*, and *R. mucilaginosa*. Although quadrupled dose of corticoids was discontinued and ulcer healing was observed, the patients required topical therapy for maintained xerostomia. Progressive systemic autoimmunity (seronegative Sjögren’s syndrome with major organ involvement) was observed later.

**Conclusion:** Topical steroids (especially in quadruple dose) require attention to safety, immunomodulation, and microbiological outcome. They showed systemic side effects: microbiome alteration, humoral (IgG) immunodeficiency, and systemic autoimmunity. Isolation of three species of *Rothia* from a patient with mouth ulcers after steroid therapy suggests their participation in infectious and inflammatory processes. The proposed a methodology using MALDI-TOF-MS may be a prototype approach for microbial diagnostics in clinical trials of immunomodulatory drugs.

## 1 Introduction

Asthma and chronic obstructive pulmonary disease (COPD) have been treated with steroids for many years. Inhaled glucocorticoids (ICS) were introduced later, initially as a topical application of hydrocortisone (London and Alexander, 1951), with higher efficacy than antihistamines or allergen-immunotherapy (Bagratuni, 1960). ICS have become the first-line treatment for asthma and sometimes COPD, because of beneficial effects in many inflammatory respiratory system diseases. On the other hand, bacterial infection of the lower respiratory tract contributes to approximately 50% of COPD exacerbations. Lung microbiome may reflect micro-aspiration of oral microbiota, but the strict role of the lung microbiome remains unidentified (Pragman et al., 2012; Zdziarski, 2020). For example, bacteria-associated exacerbation was defined as colony-forming units greater than 10^7^/ml sputum or a positive culture result (Ghebre et al., 2018), but collected microorganisms in the respiratory tract may be micro-aspiration-derived or through carryover (e.g., bronchoscopic) (Charlson et al., 2011). Although recently in pediatric (Cazeiro et al., 2017) and adult practice (Chen et al., 2020) there are ample data and meta-analyses showing the increased incidence of ICS-induced infections, they raise serious doubts. Many studies of ICS safety had reporting bias: infectious and inflammatory complications are insufficiently described with non-adequate terminology such as pneumonia, upper respiratory tract infection, etc. (Zdziarski et al., 2017a). Confirmation of infectious process with laboratory and microbiological testing was not carried out. Furthermore, in a meta-analysis, cases of serious pneumonia were defined as for hospitalization or as death from pneumonia (Yang et al., 2017). In clinical trials, drug-induced dysbiosis, secondary immunodeficiency, and opportunistic infection profile are not reported. Although the changes in the microbiome in asthmatic and COPD patients are well described, the effects of ICS have not been evaluated (Charlson et al., 2011; Erb-Downward et al., 2011; Ghebre et al., 2018). Only one study shows data with increasing caries and dental plaque in asthmatic adolescents using ICS but without strict microbiological analysis (Santos et al., 2012). Sample collection and microbiological analysis are crucial for further interpretation and conclusions. Only one recent study revealed an increased risk of oropharyngeal colonization by *Streptococcus pneumoniae* (Zhang et al., 2013), but it is not known whether such colonization should be considered as a preclinical phase of the disease or a change in the natural microbiome. However, one meta-analysis revealed a protective effect of ICS against pneumonia in patients with asthma (Bansal et al., 2015). The anti-proliferative and immunosuppressive effects of ICS, the direct effect on the respiratory epithelium, and the disruption of lung microbiome are most likely to be implicated.

The aim of the study was to describe a simple, universal, and cost-effective method of microbiome analysis for clinical trials. Such a general method for monitoring pharmacovigilance should be widely available and reliable. We were looking for the optimal method of sampling, culturing, and microbiological analysis. By trying the method in one of the patients treated with high doses of ICS, we made an unexpected finding: severe dysbiosis during induction phase of autoimmune lymphoproliferative disease, i.e., Sjögren’s Syndrome (SS).

## 2 Materials and Methods

### 2.1 Material (Case Presentation)

**FIGURE 1:**
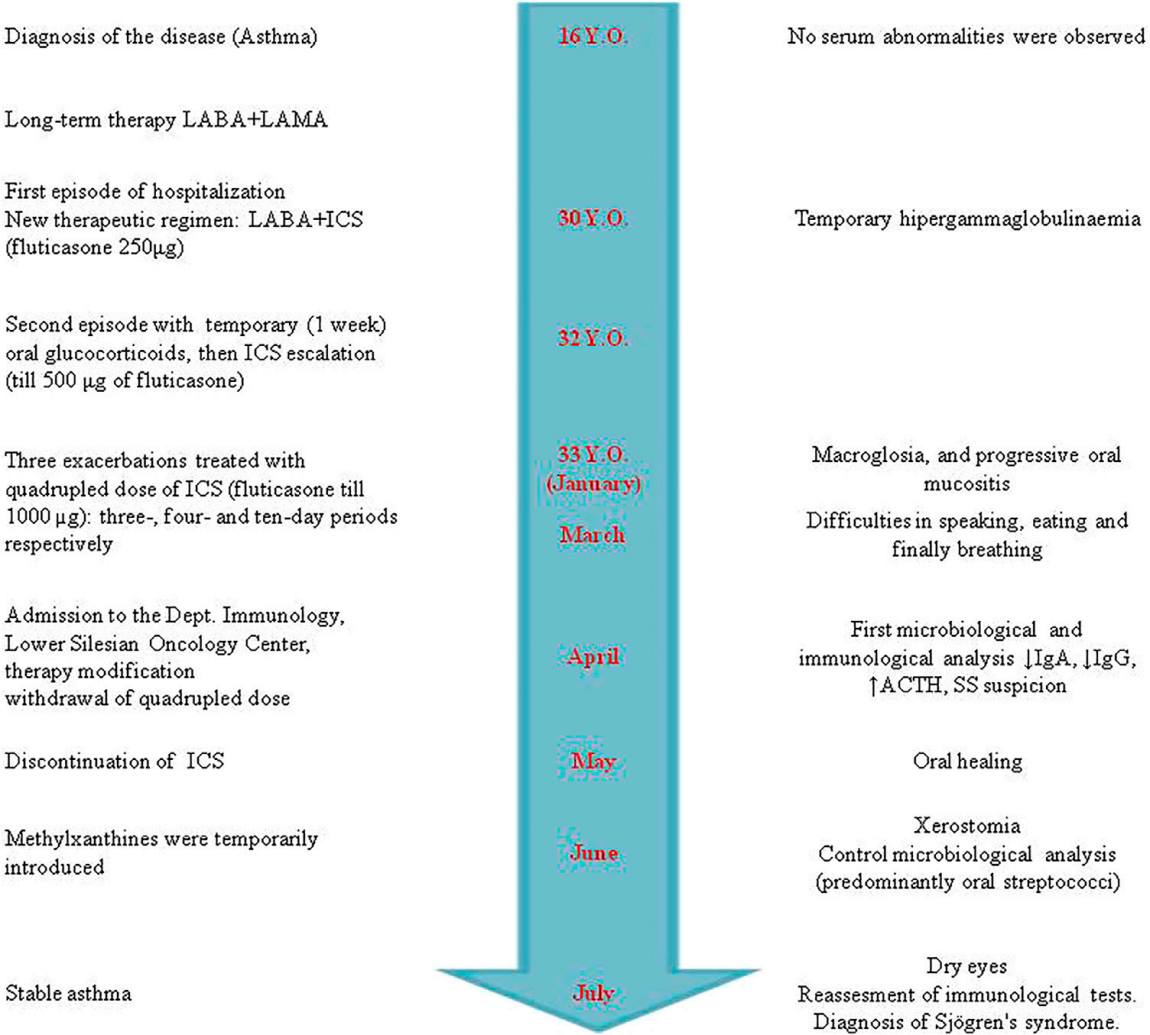
Timeline of clinical presentation, therapeutic regimen of asthma, and Sjögren’s syndrome development. The course and therapeutic interventions of asthma are presented on the left. The right side shows the sequence of symptoms and immune parameters during the development of Sjögren’s syndrome. In contrast to the similar adverse drug reactions (ADRs), i.e., drug-induced lupus (DIL), disappearance of clinical manifestation was not observed. The patient has Sjögren’s syndrome (SS) symptoms even now [more than a year and a half after the inhaled corticoid (ICS) discontinuation].

A 32-year-old, non-smoking male patient was admitted to the Department of Clinical Immunology of Lower Silesian Oncology Center in Wroclaw, Poland, with suspicion of Sjögren’s syndrome (difficulty in swallowing dry food, xerostomia, and ocular discomfort). Previously, he has been treated with a low dose of ICS (i.e., fluticasone propionate 150–250 µg/day) and long-acting beta_2_ agonists (LABA, i.e., formoterol) due to atopic asthma (Figure 1). On exertion, he was treated with high doses, up to 1,000 mcg, of fluticasone daily (quadrupled dose) and was asked to rinse his mouth with water after using the inhaler, but without the use of a spacer to reduce side effects in the mouth and throat. The patient was neither on an extreme diet, disease-modifying antirheumatic drugs, retinoids, antibiotics nor had mucositis/gastroenteritis or dental intervention for at least 2 months prior to sampling.

### 2.2 Adverse Drug Reaction Causality Assessment

The clinic-based World Health Organization–Uppsala Monitoring Center system (WHO–UMC) or Naranjo’s algorithm was used for causality assessment in type B (unpredictable) and type A (predictable) adverse drug reactions (ADRs) (Pande, 2018). The WHO–UMC scale was used, intended mainly as a convenient tool for assessing individual case. In Naranjo’s algorithm, ADR was categorized into the following four categories: ≥9 = definite ADR, 5–8 = probable ADR, 1–4 = possible ADR, and 0 = doubtful ADR.

### 2.3 Sample Collection and Growth Condition

Oral dry swab was taken with simple Viscose Swabs Applicator (Equimed, ELATALA^®^, DELTALAB S.L. SP) or with transport Amies medium without charcoal (Equimed^®^, DB S.L. SP) in the laboratory, seeded within 20 min and 6 h, respectively. In this way, it was compared whether the transport and the use of the transport medium can positively or negatively affect the microbiological result. This pre-analytical element influences the results and reporting of clinical trials, usually with the submission of materials to a central laboratory. Both swabs were cultured on solid media: blood agar, nutrient agar, tryptic soy-thioglycolate agar (Paściak et al., 2003), and brain–heart infusion (BHI) agar in duplicate to investigate different growth conditions, i.e., aerobic and anaerobic. Aerobic culture takes from 24 to 48 h at 37°C. Anaerobic conditions were obtained with the use of *GasPak EZ Anaerobe Pouch System®* (Pouchoxygen Becton, Dickinson and Company) and 5–7 days of incubation at 37°C. All different colonies were selected by two experienced microbiologists and subjected to matrix-assisted laser desorption ionization–time of flight mass spectrometry (MALDI-TOF-MS) analysis.

### 2.4 Matrix-Assisted Laser Desorption Ionization–Time of Flight Mass Spectrometry Analysis

After excising the separate colony, the standard ethanol-formic acid protein extraction method was used according to the procedure recommended by the spectrometer manufacturer (Pasciak et al., 2015). Alpha-cyano-4-hydroxycinnamic acid was used as a matrix, and MALDI-TOF-MS analysis was performed on the Ultraflex mass spectrometer (Bruker Daltonics, Germany). Spectra were recorded in the positive linear mode for a mass range of 2,000–20,000 Da and were obtained by at least 2,800 laser shots acquired from four spot positions under control of Flex Control software 3.1 (Bruker Daltonics). The spectra were externally calibrated using the *E. coli* DH5-alpha standard (Bruker Daltonics) consisting of six ribosomal *E. coli* proteins, RNase A, and myoglobin. The Biotyper 3.1 software (Bruker Daltonics) with a database containing 4,613 entries was used for strain identification. Criteria used in identification, according to the manufacturer, were as follows: a score value below 1.699 meant that the identification was unreliable, 1.7–1.999 probable genus identification, 2.0–2.299 reliable genus identification, and 2.3–3.0 highly probable species identification.

## 3 Results

### 3.1 Microorganism Identification

The clinical material was taken by the application of dry swabs with simple Viscose Swabs Applicator and transport Amies medium without charcoal. Identification of the isolates after cultivation in appropriate conditions was carried out using the MALDI-TOF Biotyper (Table 1). Comparing the aerobic and anaerobic cultures, only two species taken from dry swab were repeated (i.e., *Staphylococcus epidermidis* and *Streptococcus salivarius*), but none from the transport Amies medium. Surprisingly, we found three different species of *Rothia* genus in one patient: *R. aeria*, *R. dentocariosa*, and *Rothia mucilaginosa* in the same niche, characterized by different MALDI-TOF mass spectra. Collection of microbiota with transport swab gave a significantly narrower microbiome repertoire because strains of *S. epidermidis*, *Neisseria macacae*, *N. perflava*, as well as *R. mucilaginosa* were not detected in these conditions. The dry swab collection method and anaerobic growth conditions allowed us to detect a significantly higher abundance of oral microbiota, but without *Rothia* spp*.* (Table 1).

**TABLE 1:** MALDI-TOF-MS Biotyper identification of microorganisms.

Cultivation conditions^a^	Dry swab^b^	Transport amies medium^c^
Aerobic	*Staphylococcus epidermidis* (2.270)	*Streptococcus sanguinis* (2.127)
	*Streptococcus salivarius* (2.23)	*Streptococcus salivarius* (2.268)
		*Streptococcus oralis* (2.133)
	*Neisseria macacae* (2.048)	
	*Neisseria perflava* (2.384)	
	*Neisseria flavescens* (2.368)	*Neisseria flavescens* (2.277)
	*Neisseria mucosa* (1.835)	*Neisseria mucosa* (2.518)
	*Rothia aeria* (2.165)	*Rothia aeria* (2.176)
	*Rothia dentocariosa* (1.969)	*Rothia dentocariosa* (2.518)
	*Rothia mucilaginosa* (2.473)	
Anaerobic	*Staphylococcus epidermidis* (2.24)	
		*Streptococcus mitis* (2.300)
	*Streptococcus pneumoniae* (2.294)	*Streptococcus pneumoniae* (2.294)
	*Streptococcus salivarius* (2.349)	
	*Streptococcus sanguinis* (2.263)	
	*Streptococcus parasangunis* (2.107)	
	*Streptococcus australis* (1.747)	
	*Propionibacterium sp* (2.026)	
	*Propionibacterium acidifaciens* (1.776)	
	*Propionibacterium acnes* (1.991)	
	*Actinomyces odontolyticus* (2.126)	
	*Actinomyces oris* (2.29)	

Microbiota from the patient were cultivated in aerobic and anaerobic conditions (the same clinical sample) taken parallel on a dry swab and transport medium, respectively. Score values are presented in brackets.

a Cultivate conditions: aerobic—24–48-h incubation on solid medium—blood agar, BHI agar, or nutrient agar at 37°C; anaerobic—5-day incubation on solid medium blood agar, brain–heart infusion (BHI) agar, or tryptic soy thioglycollate agar at 37°C in jars with use of Gas-Pack system (GasPak EZ Anaerobe Pouchoxygen Becton, Dickinson and Company© with O_2_ ≤1% and ≥10% CO_2_ as described in product details.

b Sample from dry swabs were collected with simple Viscose Swabs (Equimed®, DELTALAB S.L. SP)

c With transport Amies medium Equimed® (DELTALAB S.L. SP).

### 3.2 Clinical Sequel

**FIGURE 2:**
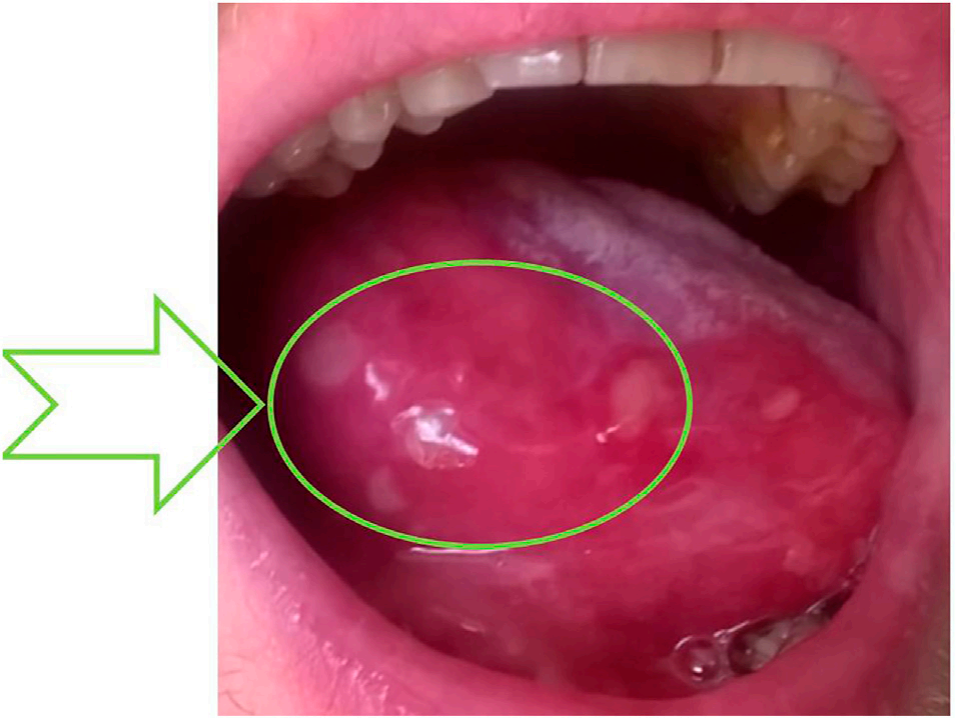
WHO stage 3 of oral mucositis caused by dysbiosis after quadrupling fluticasone therapy. Macroglossia—enlargement of the tongue with acute epithelial disruption with fibrin, called epithelitis—and many ulcers were observed as a sign of lymphocytic infiltration of the epithelia. Formation of a fibrin coating—the place of subsequent sampling (dysbiosis state) —is indicated by an arrow. The probable ADRs are reported as L453/2020 in Polish Pharmacovigilance Service, collectively with data presented in the upper part of Table 2.

Because of mucositis, dysbiosis state, and clinical manifestation of adverse ICS reaction (Figure 2), the immunoparameter analysis was performed (Table 2). Although a quadrupled dose was not continued over the 10-day period (Figure 1), insignificant adrenocortical suppression was observed (i.e., with a mild increase of ACTH) and a slight decrease of serum IgG and IgA. Iron deficiency was not observed (Table 2). Further immunological analysis excluded primary immunodeficiency, especially deficiency of IgA, which was at a normal level in blood, but a quantitative deficit of saliva was later observed. Physical examination showed no significant eye dryness (i.e., Schirmer’s test 10 mm in 5 min) nor any further abnormalities, but submandibular lymph glands were slightly swollen and oral mucosa showed WHO stage 3 of oral mucositis and 3–8 mm erosion/ulcers and a bitter taste on the back of the tongue (Figure 2). Hypertrophy of the tongue (macroglossia) with consequences and malfunction of the upper respiratory tract caused breathing and speech problems. Tongue base-induced obstructive sleep apnea was observed. After the withdrawal of ICS in quadrupled dose, the oral ulcer disappeared, but prolonged WHO stage 2/3 of oral mucositis was observed. It required topical therapy with mucoprotectants, drugs usually used for chemotherapy-induced oral mucositis. The duration of quadrupled treatment was as short as possible and was not continued after the symptoms disappeared (Figure 1). Although higher doses were not continued over a 10-day period, insignificant adrenocortical suppression was observed (i.e., with mild increase of ACTH and sodium retention) as well as secondary immunodeficiency (i.e., weak decrease of serum IgG level and destruction of mucosal barrier with epithelitis) (Table 2). After discontinuation of ICS, the oral mucositis prolonged as mild signs of seronegative primary Sjögren’s syndrome with major organ involvement (Tezcan et al., 2017). Three months later, the patient showed no serum abnormality, but prolonged lymphadenopathy, splenomegaly, saliva deficiency, and positive autoantibody such as rheumatoid factor, SS-A, were observed (Figure 1; Table 2). On the basis of the clinical presentation and formal criteria, which require the presence of immunologic abnormalities (i.e., SS-A antibody or lymphocytic infiltration in labial salivary gland), the SS diagnosis was made (Sandhya et al., 2017). After ICS discontinuation, the patient did not develop asthma exacerbation within 6 months (Figure 1). The *Rothia* spp. diversity was not observed later—streptococcal growth predominated (Figure 1). Contrary to the first microbial analysis (presented in Table 1), the second one revealed *S. salivarius*, *Streptococcus oralis*, *S. pneumoniae*, *Neisseria* spp., as well as *Candida albicans*. However, this could be due to the inflammatory response, changes in immunity, and the microbiome seen in Sjögren’s syndrome as described previously (Almståhl et al., 2003).

**TABLE 2:** Laboratory data of patients with quadrupling inhaled glucocorticoid (i.e., fluticasone 1,000 µg/day) and patient’s characteristics.

Parameter	Initial (May)	Normal value	Control visit 3 months later (July)	WHO-UMC causality term^b^ (Naranjo’s score^a^)
ACTH (at 7 a.m.)	40→55 mg/L = 11µMol	<50	38	Certain
				Definite (10)
Ferrum (Fe)	15 mM/l	10–40	NA	NA
IgG	750 mg/dl	800–1,600	1,100	Certain
				Definite (10)
IgM	169 mg/dl	34.0–210.0	135	NA
IgA	77 mg/dl	88.0–410.0	92	Certain
				Definite (10)
CRP	11.8 mg/l	0.00–5.00	NA	NA
K^+^	3.65 mM/l	3.5–4.5	4.0	Possible
				Possible (4)
Na^+^	148 mM/l	137–143	142	Certain
				Definite (10)
Cl	112 mM/l	100–110	115	NA
HBA_1c_	5%	<6.5%	4%	NA
Anti-SS-A (Ro)	Negative	—	Positive	Possible
Anti-SS-B (La)	Negative	—	Negative	NA
RF (rheumatoid factor)	Negative	—	Positive (IgG class)	Possible
Anti-dsDNA	Negative	—	Negative	NA
Schirmer’s test (wetting of the paper after 5 min)	10 mm	>15 mm	9 mm	Possible
Spleen length	13 cm	<12 cm	16 cm	Possible

a The upper part of the table shows the biochemical/laboratory parameters that are part of the predictable ADRs and therefore allow the use of the WHO-UMC system and Naranjo’s algorithm. The causality for ICS was assessed following prior epidemiologic data, comparison initial, and control visit.

b The lower part of the table presents the serological and leading parameters (criteria of Sjögren’s syndrome) as category B ADRs, classified according to the WHO-UMC system (qualitative data) and reported separately (L452/2022).

### 3.3 Causality Assessment

The clinical course to a limited extent allowed for unambiguous answers in individual parts of the questionnaires. Some symptoms can be attributed to the increased dose (quadrupled) and some to steroids. The change in ACTH (and other biochemical/laboratory parameters presented in Table 2) is an indirect indicator of ICS toxic concentrations. Basing on prior epidemiologic studies (prior knowledge) and information obtained from a given case (especially dechallenge), these ADRs were classified as predictable and certain according to the WHO-UMC system. Although rechallenge was difficult to perform ethically (especially in quadrupled dose of ICS), discontinuation of ICS (dechallenge) corresponded with oral reconstitution (Figure 1) without *Rothia* overgrowth. Furthermore, dysbiosis and macroglossia were in strict time relationship with quadrupled dose (onset in January), and the response to withdrawal was observed (May) (Figure 1), and the ADRs was classified as probable. The answer to the survey question in Naranjo’s algorithm (Was the mucositis more severe when the dose was increased?) is positive in the first period (i.e., when the patient escalated their ICS doses). However, the follow-up question (Was the mucositis less severe when the dose was decreased?) is not so clear after ICS withdrawal (SS prolonged). Therefore, oral mucositis may be classified as probable or possible according to the WHO-UMC system. Formoterol (LABA) and temporary short-acting beta-agonists (e.g., salbutamol) were still used; therefore, the ADRs were unlikely the cause of biochemical parameters Table 2.

## 4 Discussion

### 4.1 Microbiological Methods of Microbiota Analysis and Methodological Difficulties

The studies of the human microbiome have revealed that healthy individuals differ remarkably in the oral cavity microbiota composition. Based on the analysis of 2,589 16S rRNA clones, the bacterial diversity of the microflora from nine different sites of five clinically healthy subjects revealed the genus *Rothia* among many others (Aas et al., 2005). In further study of 10 healthy human individuals, Bik et al. (2010) found that the genus *Rothia* was abundant in the oral cavity and was present in all 10 individuals. However, *Rothia* spp. are not described as a typical commensal in “Structure, function and diversity of the healthy human microbiome” (Human Microbiome Project Consortium, 2012). Microbiome encompasses the microbiota and its host environment, but the latter is rarely included in the analysis (Zdziarski, 2020; Raffatellu and Bäumler, 2021; Zdziarski et al., 2017b). In other words, there is no “healthy” microbiome (Yong, 2014), and an integrated approach is crucial (Brinker et al., 2019). Interestingly, higher abundance of *R. mucilaginosa* was observed previously in periodontal patients (Camelo-Castillo et al., 2015), but another publication shows the three most active microbial players, e.g., *Porphyromonas gingivalis*, *Treponema denticola*, and *Fusobacterium nucleatum* (Deng et al., 2018)*.* Noteworthy, in several case reports (the description of four *Rothia* species is the last decade’s finding), the isolation of only one species of *Rothia* predominates (Falcone et al., 2012; Zhou and Li, 2015). Our observation of three different *Rothia* species in the same sample from the patient is the first in the literature, regarding the described tissue and disease conditions. Appropriate specimen collection and storage before arrival at the molecular diagnostic laboratory are crucial (Huse et al., 2014). Furthermore, DNA/RNA false-negative results are minimized by avoiding the use of swabs with wooden shafts or cotton tips (the swab that has been validated for the amplification assay must be used). It prompts the use of a dry swab (without transport medium) or brush sampling method rather than lavage or biopsy in microbiome sampling as described elsewhere for the assessment of gut microbiota (Huse et al., 2014). The concerns raised above indicate that the pre-analytical stage of sample management is crucial to get credible results. However, the first and second elements of the diagnostic chain seem to be crucial for the final result (Table 1), which is partly the answer why the composition of the oral microbiome in various publications is so diverse (e.g., Human Microbiome Project Consortium, 2012; Zhou and Li, 2015). An identification method using MALDI-TOF-MS turned out to be very efficient in identification down to the species level. This is crucial because identification at the genus level is insufficient for proper classification (Nouioui et al., 2007). Therefore, without strict uniform diagnostic chain, it is impossible to define a healthy microbiome by itemizing microbial species or cataloguing their genes (Zdziarski 2020; Raffatellu and Bäumler, 2021).

Based on our observations, we propose a widely available and reliable diagnostic chain for monitoring pharmacovigilance that should consist of the following:

• Sample collection (near laboratory) with dry swab (transport medium is not useful—Table 1)
• Aerobic as well as anaerobic (Gas-Pack) culture of microorganisms (various media)
• Identification with MALDI-TOF-MS
• ADR reporting and terminology (dysbiosis, macroglossia, immunodeficiency, and secondary inflammatory disease)
• Modification of therapeutic regimen (e.g., microbiota transplantation, pre–pro-biotics, and ICS withdrawal), nomenclature, and guidelines, e.g., the Global Initiative for Asthma (2019).

### 4.2 Clinical Repercussion in Therapeutic Regimen

Our data may be important for patients with long-term oxygen therapy as well as for storing and transporting of clinical material to the laboratory (i.e., with access to oxygen). In hypoxic patients, shifts and changes in the microbiome and alpha diversity may occur when oxygen therapy (or the use of oxygen for ICS nebulization) is administered, similar as presented in Table 1. Charlson et al. (2011) suggest that the lung microbiome reflects micro-aspiration of the oral flora. The risk of microbial transition is much higher after aerosol delivery, especially ICS (O’Malley, 2015). Risk factors for *R. mucilaginosa* bacteremia include prolonged and profound neutropenia, malignancy, and an indwelling vascular foreign body. Unfortunately, most of the literature indicates the risk factor, but the retrospective study identified no qualitative or statistically significant differences between the two groups for any of the variables collected, including recent corticosteroid use (6% versus 11%, *p* = 1.0) and the presence of neutropenia (88% versus 89%, *p*= 1.0) (Ramanan et al., 2014).

### 4.3 DATA Collection and Discrepancy: Non-Adequate Nomenclature for Infectious Complications After ICS

There are major challenges in specifying relevant outcomes and study designs for evaluating adverse drug reactions (Zdziarski et al., 2017a). High diversity in reporting, as well as variation in their definition, methods of ascertainment, and grading, is an important problem in clinical studies (Peryer et al., 2020). One of the crucial limiting factors in clinic-based causality assessment in clinical immunology practice is the long latency of many immunomediated ADRs (Zdziarski, 2019) as presented previously for anaphylaxis: IgE-mediated (type 1—“immediate”) allergic reactions were observed 5 or 14 days after drug administration (Zdziarski et al., 2017a). Naranjo’s algorithm was not useful (Pande, 2018). In our observation, it is difficult to unambiguously associate biochemical changes with the chronic use of ICS or a quadrupled dose, and there may have been an accumulation of them (Figure 1; Table 2). On the other hand, macroglossia and mucositis are symptoms directly and continuously observed by the patient, and the time relationship is clearer (Figure 1). Although oral mucositis is prolonged, the type of inflammation (bacterial to autoimmune) and the clinical picture (i.e., presented in Figure 2 to dry mucositis in SS syndrome) changed. The Common Terminology Criteria for Adverse Events (CTCAE) and Naranjo’s algorithm do not provide for such specific and qualitative scenario. Information taken from published reports may be incomplete or may lack specificity because of usually observed differences in coding and categorization of adverse effects between studies (Pande, 2018). Most of the studies do not differentiate between adverse event (harmful outcome that occurs, not necessarily caused by a drug) and adverse effect (causal relation between the drug and the event is at least a reasonable possibility). Following CTCAE, the infectious complications are, therefore, described usually as localized or life-threatening colitis, pneumonia, etc., which is an inflammatory rather than a strictly infectious process. For example, the last meta-analysis of 17 randomized controlled trials (20,478 patients) showed a significantly increased risk of upper respiratory tract infections in COPD patients with ICS therapy (Chen et al., 2020). These studies were dedicated to assessing the efficacy and safety of ICS treatment rather than the infectious profile. Upper respiratory tract infection (URTI) and pneumonia were not accurately defined as an infection without microbiological analysis, species identification, or at least type (opportunistic vs. pathogen-related). For example, there were eight deaths among patients from pneumonia as such in the combination therapy group, seven in the placebo group, nine in the salmeterol group, and 13 in the fluticasone group. Surprisingly, the URTI rate is higher in the combination therapy group than in the placebo group and in patients with fluticasone monotherapy (i.e., 0.11, 0.1, and 0.09 rate per year, respectively) (Calverley et al., 2007). The infection is, therefore, difficult to link with ICS (incidence rate lower than placebo), especially endogenous and opportunistic infections. Moreover, the meta-analysis corresponds with our report:

• Fluticasone was observed with an increased risk of URTI in comparison with other ICS (e.g., mometasone);
• High-dose fluticasone treatment was associated with a significantly higher risk of URTI but not low dose.

Our observation indicates the need for the implementation of microbiological testing and species identification in the coding and reporting of adverse effects in clinical trials. Identification using MALDI-TOF-MS as a relatively inexpensive and increasingly accessible method should be the standard. The species diversity of the cultured bacteria (Table 1) indicates that the dysbiotic state precedes the subsequent general symptoms and should, therefore, be described as a separate category or at least a separate grade in infectious complications in CTCAE. Regretfully, clinical trials of inhalation drugs, as well as all therapeutic regimens, do not implement microbiome analysis.

#### 4.3.1 Patient Risk Factors and ICS: Friend or Foe?

Although *Rothia* were described as health-associated genera, these bacteria can cause disease in severe immunodeficiency. *Rothia* spp. are increasingly being recognized as emerging opportunistic pathogens (Abidi et al., 2016). Our observation shows the clinical repercussion of inhalators, especially steroid overuse. Unfortunately, routine checks for microbial colonization and surveillance cultures from patients are not recommended (O’Malley, 2015), but asthma exacerbation is frightening for a patient, and self-management of ICS prompts overdosing. The concept and strict plan for patients, which included a temporary quadrupling of the ICS dose, were described previously (McKeever et al., 2018). The finding showed five events of severe pneumonia (0.5%) with one death in the quadrupling group of 957 patients. Our findings (Table 2) indicate that inhaled corticoids are absorbed with systemic side effects. Furthermore, the post-ICS epithelitis and macroglossia (Figure 2) caused breathing perturbation. The oral cavity can act as the site of origin of dissemination of pathogenic organisms to distant body sites in immunocompromised hosts, especially those suffering from malignancies, diabetes, and rheumatoid arthritis, or SS immunosuppressive treatment. Only one study shows lung microbiome alteration in patients treated with ICS (Pragman et al., 2012). Acquired causes of macroglossia may include endocrine disorders and inflammatory or infectious diseases. However, this rare symptom has not been described in more detail, especially in the light of ADRs and the microbiome as well as endocrine and immune system disorders (Genetic and Rare Diseases Information Center, 2022).

#### 4.3.2 Inhaled Glucocorticoid-Induced Immune Perturbation

**FIGURE 3:**
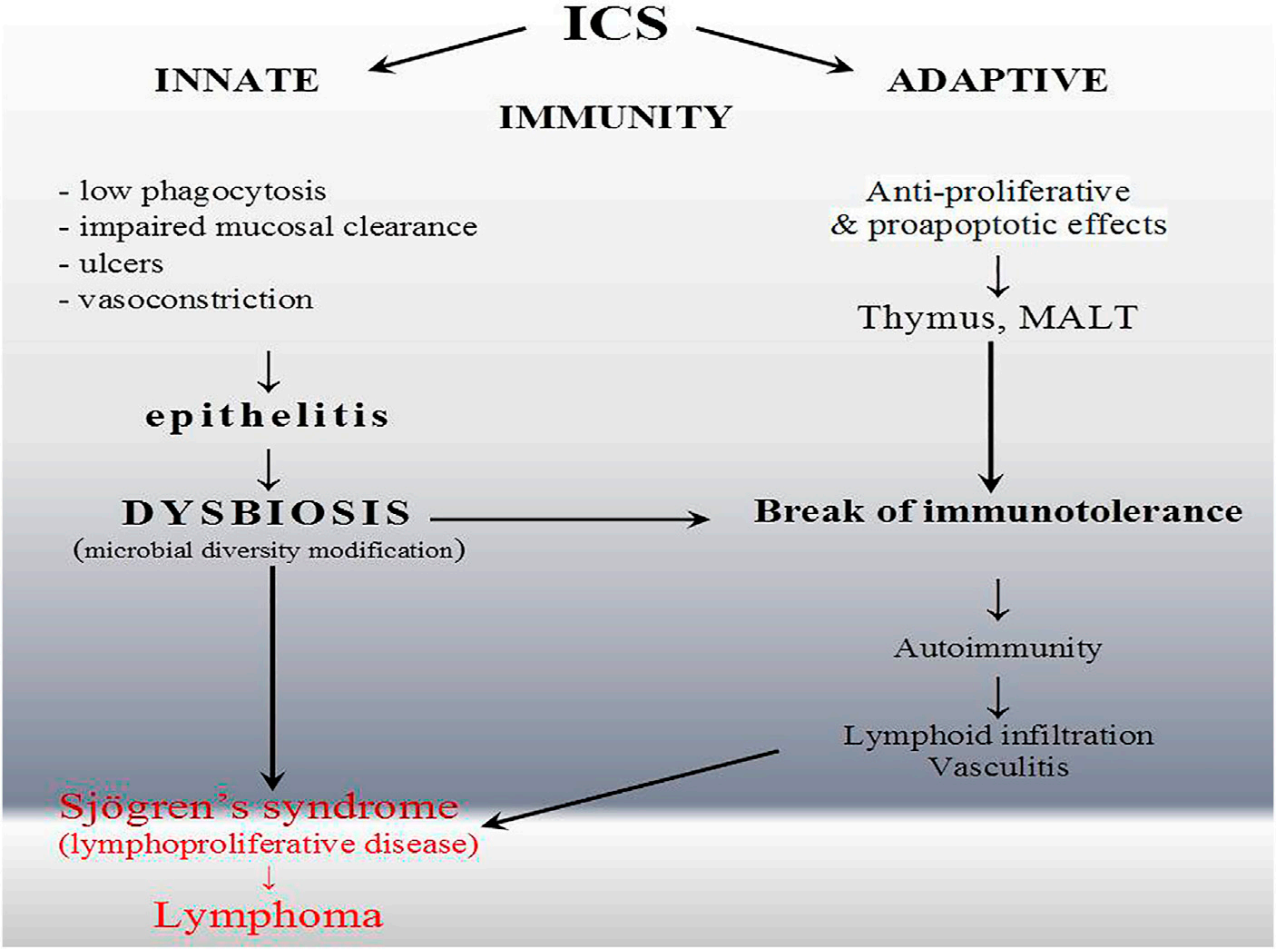
Inflammatory complication of inhaled corticoid (ICS) overuse. The wide spectrum of immunomodulatory effects of ICS and influence on epithelial barrier prompt lymphocyte selection in thymus (T cell) or mucosa-associated lymphatic tissue (MALT) (B and T cells) intensified by non-specific perturbation and dysbiosis. ICS, unlike drugs typically associated with drug-induced lupus, have a direct influence on the immune system. Nevertheless, they also induced systemic rather than organ-specific autoimmunity.

Innate immune response is disturbed in our patient (Table 2) (Bishop and Gleeson, 2009). Our observation corresponds with the data that only high-dose ICSs are associated with a significantly increased risk of infection of the upper respiratory tract but preferentially endogenous with dysbiosis (Figure 3). Furthermore, invasive or recurrent pneumococcal diseases were observed only in immunodeficient child with antibody deficiency (Butters et al., 2019). The ICS-induced transient humoral immunodeficiency observed here requires reflection. Until now, ICS has not been described as a risk factor for *Rothia* opportunistic complication. This is the first description and, what is noteworthy, of three different species simultaneously in the same niche. The genome-based taxonomic classification of the phylum Actinobacteria as well as *Rothia* spp. is still an open issue (Nouioui et al., 2007). We hypothesize that alterations in the oral mucosa microbiome and/or its interactions with the host immune system (e.g., low IgG, lymphadenopathy, and splenomegaly (Table 2; Figure 3) may lead to disordered immune tolerance and the development of an inflammatory state that accelerates the progression of asthma as well as induces autoimmune-lymphoproliferative disorder, i.e., Sjögren’s syndrome (Figure 3). Unfortunately, strong alloantigenic stimulation by microbiome and narrow lymphocyte repertoire prompt lymphoproliferative disease (Zdziarski et al., 2017c). T cell receptor (TCR) threshold activity leading to such drastically opposing signaling outcomes (life or death) is modulated in part by glucocorticoids (Figure 3) (Wiegers et al., 2011). Dysbiosis and the role of microbiota in Sjögren’s syndrome clinical presentation are the area for further research. Endogenous glucocorticoids, to some extent blocked by ICS with the increase of ACTH (Table 2), are required for a robust adaptive immune response because of their promotion of the selection of T cells, and the absence of thymocyte glucocorticoid signaling results in an immunocompromised state with alterations in the TCR repertoire of polyclonal T cells (Mittelstadt et al., 2012). Only through an integrated approach that considers influences of multiple interacting factors we will be able to gain a better understanding of host–microbe associations (Brinker et al., 2019; Zdziarski, 2020).

#### 4.3.3 Sjögren’s Syndrome Pathogenesis and Overlapping With Asthma/COPD

Sjögren’s syndrome is a chronic autoimmune disease that classically affects the lacrimal and salivary glands and can affect almost any organ system in the body including the lungs (Sandhya et al., 2017). Lung involvement in primary SS is mainly related to the small airway disease (Papiris et al., 1999). Intriguingly, even in SS, the gut microbiome is more likely to be studied than the respiratory one, as reviewed elsewhere (Tsigalou et al., 2018). It revealed significant compositional differences compared to controls, while *Firmicutes/Bacteroidetes* ratio and *Actinobacteria* decreased (Moon et al., 2020). A similar observation was presented in our case. As SS developed, a wide variety of microbes (Table 1) became dominated by streptococci. Whether it is an effect or a cause, most studies are not conclusive, and indirect pieces of evidence are the only elements in this puzzle thus far (Tsigalou et al., 2018). MALT activation in preclinical phase of SS overlaps with inflammatory symptoms, and clinical presentation of inflammatory respiratory diseases usually treated with ICS (Zdziarski and Gamian, 2019), bronchial hyper responsiveness, cough, and bronchiolitis or bronchiectasis, are reported in SS with a prevalence between 7% and 61% (Flament et al., 2016). There are currently no SS treatments available that address the underlying disease etiology, and systemic or topical steroids are not effective. Initial autoimmune inflammation and epithelitis—a deregulated immune response—are the first phase of SS development (Tapinos et al., 1999) (Figure 2). The overuse of ICS and quadrupling of therapeutic regimen in asthma may be paradoxically the source of microbial dysbiosis, systemic inflammatory complication, and systemic autoimmunity (relatively rare seronegative SS with major organ involvement). The lack of hyper-gammaglobulinemia and low IgG observed here show that patients receiving ICS may develop secondary immunodeficiency as well as atypical SS presentation (Table 2). In our observation, dysbiosis and *Rothia* spp. overgrowth were not observed after ICS withdrawal (patient recovers well after ICS stopping), but contrary to early immunoabnormalities (top of Table 2), late immunoabnormalities and SS are difficult for causality assessment (qualified as possible as presented in the bottom part of Table 2). ICS show extraordinary pleiotropic effects, and SS is an unknown-etiology disease (Figure 3).

### 4.4 Limitations

Our research had several limitations. Firstly, the baseline samples were not collected prior to treatment initiation. The study was not prospective, and the method and diagnostic chain were presented in one, which is the most transparent case. It would be very difficult to obtain patients with a newly diagnosed disease, then to observe them with waiting for a similar situation and a quadrupling ICS dose. Besides, it would never be certain whether the starting sample is a native microbiome or disease altered (e.g., asthma). Secondly, the limitation of the presented diagnostic chain is transport and culture (Table 1). These do not allow the detection and identification of organisms that have not been cultured in the laboratory, e.g., Archaea, which are involved in periodontal disease (Vianna et al., 2006). Thirdly, there is no simple and direct evidence that the observed therapy and microbiome are the direct cause of the development of Sjögren’s syndrome, a relatively rare autoimmune disease. However, apart from classical rheumatic fever and Group A streptococcal infection, the early stages of autoimmune diseases (the primary immune response) and the induction phase of the disease are not known. This accidental finding in the later observation of the patient, however, may be a sufficient example of the undiscovered role of the respiratory microbiome. Most of the studies, including in Sjögren’s syndrome, are of the gut microbiome (Tsigalou et al., 2018).

## 5 Conclusion

Our findings shed a new light on an adverse effect of ICS and the initial phase and possible pathogenesis of Sjögren’s syndrome. ICS and their overuse or quadrupling prompt oral and respiratory tract microbiome discrepancy (dysbiosis) and secondary immunodeficiency; therefore, opportunistic infections with microorganisms such as from the genus *Rothia* are erroneously omitted. Such dysbiosis should be considered a preclinical phase of the disease, and an area for further research should be provided. Identification with MALDI-TOF-MS as a cheap and increasingly accessible method may be a prototype approach.

## Data Availability

The datasets presented in this study can be found in online repositories. The names of the repository/repositories and accession number(s) can be found below: All data generated or analyzed during this study are included in this article. The clinical isolate was deposited in a publicly accessible culture collection—Polish Collection of Microorganisms.

## Abbreviations

ACRH, adrenocorticotropic hormone; ADRs, adverse drug reactions; AE, adverse event; COPD, chronic obstructive pulmonary disease; CTCAE, Common Terminology Criteria for Adverse Events; ICS, inhaled glucocorticoids; LABA, long-acting beta2-agosts; MALDI-TOF-MS, matrix-assisted laser desorption ionization–time of flight mass spectrometry; MALT, mucosa-associated lymphoid tissue; SS, Sjögren’s syndrome; TCR, T cell receptor; URTI, upper respiratory tract infection.
